# Bioluminescence Imaging of DNA Synthetic Phase of Cell Cycle in Living Animals

**DOI:** 10.1371/journal.pone.0053291

**Published:** 2013-01-03

**Authors:** Zhi-Hong Chen, Rui-Jun Zhao, Rong-Hui Li, Cui-Ping Guo, Guo-Jun Zhang

**Affiliations:** 1 Breast Center, Cancer Hospital of Shantou University Medical College, Shantou, People’s Republic of China; 2 Heilongjiang Province Key Laboratory of Cancer Prevention and Treatment, Mudanjiang Medical University, Mudanjiang, People’s Republic of China; 3 Cancer Research Center, Shantou University Medical College, Shantou, People’s Republic of China; National Institutes of Health, United States of America

## Abstract

Bioluminescence reporter proteins have been widely used in the development of tools for monitoring biological events in living cells. Currently, some assays like flow cytometry analysis are available for studying DNA synthetic phase (S-phase) targeted anti-cancer drug activity *in vitro*; however, techniques for imaging of *in vivo* models remain limited. Cyclin A2 is known to promote S-phase entry in mammals. Its expression levels are low during G1-phase, but they increase at the onset of S-phase. Cyclin A2 is degraded during prometaphase by ubiquitin-dependent, proteasome-mediated proteolysis. In this study, we have developed a cyclin A2-luciferase (CYCA-Luc) fusion protein targeted for ubiquitin-proteasome dependent degradation, and have evaluated its utility in screening S-phase targeted anti-cancer drugs. Similar to endogenous cyclin A2, CYCA-Luc accumulates during S-phase and is degraded during G2/M-phase. Using Cdc20 siRNA we have demonstrated that Cdc20 can mediate CYCA-Luc degradation. Moreover, using noninvasive bioluminescent imaging, we demonstrated accumulation of CYCA-Luc in response to 10-hydroxycamptothecin (HCPT), an S-phase targeted anti-cancer drug, in human tumor cells *in vivo* and *in vitro*. Our results indicate that a CYCA-Luc fusion reporter system can be used to monitor S-phase of cell cycle, and evaluate pharmacological activity of anti-cancer drug HCPT in real time *in vitro* and *in vivo*, and is likely to provide an important tool for screening such drugs.

## Introduction

The DNA synthetic phase (S-phase) of the cell cycle, during which DNA synthesis occurs, plays an important role in cell proliferation [1], [2]. Many anti-cancer drugs such as DNA topoisomerase I (Topo I) inhibitors target the elongation step of DNA synthesis, and arrest tumor cells in S-phase, subsequently induce apoptosis [3], [4]. These DNA synthesis-targeting anti-cancer drugs belong to the S-phase-specific drug class of cell cycle specific agents (CCSA), and they have been widely used clinically to treat cancer patients [5].

It is well known that drug screening is a key step in the drug discovery and development process [6]. A number of pharmaceutical companies are currently developing S-phase-specific drugs as potential anti-cancer agents; however, ideal in *vivo* tumor models for *in vivo* drug screening remain limited. Parameters in traditional tumor xenograft models such as tumor weight or volume are used predominantly to evaluate drug action. These conventional physical measurement techniques require animal sacrifice and prevent real-time monitoring of tumor progression and effects of anti-cancer drugs in living animals. They are not readily adaptable to meet requirements for high-throughput drug screening. These limitations have hampered anti-cancer drug development [7]. New *in vivo* technologies for screening S-phase-specific anti-cancer drugs are urgently needed.

During the past several years, novel bioluminescent *in vivo* imaging systems have been developed, which offer sensitive and noninvasive techniques for rapid, real-time monitoring of biological events in living cells [8]. In comparison with a traditional tumor xenograft model, these techniques have been demonstrated to be particularly well suited for monitoring tumor progression and for evaluating the effects of anti-cancer drug treatments in living animals [9]. For example, the firefly luciferase (Luc) protein is the most widely used reporter protein for noninvasive and quantitative monitoring of pharmacological activity of anti-cancer drugs such as cell cycle nonspecific agents (CCNSA) [10]. However, Luc protein is not well suited as a pharmacological reporter assay for CCSA screening in living cells because it is not regulated by cell cycle pathways, and cannot be used directly as a biological marker to monitor particular cycle-related molecular targets or pathways. Recently, several studies have demonstrated that it is possible to genetically reengineer bioluminescent or fluorescent fusion proteins to respond to specific molecular processes [11]. For example, a p27-Luc fusion protein can be used to monitor Cdk2 activity *in vitro* and *in vivo* [12]. As well, bioluminescence imaging of inhibition of IκBα-specific proteasomal degradation using an IκBα-FLuc fusion reporter can monitor in real time nuclear factor NF-κB activation and pharmacological modulation [13]. Such reporters provide excellent tools for studying and imaging molecular pathways of tumorigenesis and tumor progression, and for monitoring the pharmacological effects of molecular target-based anti-cancer drugs *in vitro* and *in vivo*.

Cyclin A2 is a cell cycle control protein that plays an important role in cell proliferation and cancer [14]. In mammalian cells, cyclin A2 levels are low during G1-phase but increase at the S-phase progression during which cyclin A2 contributes to the stimulation of DNA synthesis. Cyclin A2 is degraded during prometaphase by proteasomal ubiquitin-dependent proteolysis. The APC/C (anaphase-promoting complex/cyclosome) is responsible for coordinated waves of protein degradation that ensures proper progression through the cell cycle in a unidirectional and irreversible manner [15]. Therefore, cyclinA2 can be considered an S-phase specific protein, and can be used as a biological marker to monitor S-phase. Accordingly, we have designed a strategy in which luciferase is fused to the C-terminus of cyclin A2, forming a cyclin A2-luciferase (CYCA-Luc) fusion protein. We have further evaluated the utility of this construct for screening S-phase targeted anti-cancer drugs. Our results indicate that this CYCA-Luc fusion reporter system can be used to monitor pharmacological activity of S-phase-specific anti-cancer drug HCPT in real time *in vitro* and *in vivo*, and is likely to provide an important tool for screening such drugs.

## Materials and Methods

### Antibodies, Chemicals, Animals and siRNA

Goat polyclonal antibody against firefly luciferase and mouse polyclonal antibody to Cdc20 were purchased from AbCam (Cambredge, USA). Goat polyclonal antibody against p27 and goat polyclonal antibody against cyclin A2 were purchased from R&D (Minneapolis, USA). Mouse polyclonal antibody to β-actin was purchased from Santa Cruz Biotechnology (Santa Cruz, USA). D-Luciferin, nocodazole, and mimosine were purchased from Sigma (St. Louis, USA). 10-hydroxycamptothecin (HCPT) and paclitaxel were purchased from Huangshi Feiyun Pharmaceutical Co. Ltd (Huangshi, CN). BALB/c nude mice, 4–6 weeks of age (15–20 g), were purchased from the Animal Center of Shanghai Institutes for Biological Sciences (Huangshi, CN).

siRNAs were synthesized from GenePharma (Shanghai, CN). siRNA sequences were unique to their intended targets, based on BLAST searches. The siRNA specific to Cdc20 were 5′-GGGAAUAUAUAUCCUCUGUTT-3′ (Sense), 5′-ACAGAGGAUAUAUAUUCCCTT-3′ (Anti-Sense). The scramble siRNA were 5′-UUCUCCGAACGUGUCACGUTT-3′ (Sense), 5′-ACGUGACACGUUCGGAGAATT-3′ (Anti-Sense). All siRNAs were transfected at 100 nM final concentrations using Lipofectamine 2000 transfection agent (Invitrogen, Camarillo, USA) according to the manufacturer’s recommendations.

### Vector Construction

To make cyclin A2 and luciferase fusion construct, we PCR-amplified human cyclin A2 cDNA with the primers 5′-GCGCAAGCTTGCCACCATGTCAAACGTG-CGAGTGTCTAAC-3′ and 5′-GCGCCCATGGTCGTTTGACGTCTTCTGAGGC-CAGG-3′. The resulting PCR product was cut with Hind III restriction enzyme and ligated into the pGL3-control plasmid (Promega), in which firefly luciferase cDNA is under control of the SV40 early promoter. Final construct was verified by automated DNA sequencing.

### Stable Transfection, and Cell Cycle Analysis

#### Stable transfection

Cells were maintained in Dulbecco’s Modified Eagle Medium (DMEM) supplemented with 10% FBS. Plasmid or siRNA transfections were performed with Lipofectamine 2000 (Invitrogen, Camarillo, USA) according to the manufacturer’s instructions. To make stable cell lines, cells were cotransfected with 5 µg of pGL3-control or pGL3-CYCA-Luc and 0.5 µg of empty pcDNA3.1 (Invitrogen). Forty-eight hours later, cells were placed in medium containing G418 (1000 µg/mL) to antibacterically select transfected cells to grow.

#### Cell synchronization

U2OS cells stably expressing CYCA-Luc were blocked in late G1-phase by incubation in medium containing 0.2 mM mimosine for 20 h. Cells were then released into mimosine-free medium and were subsequently blocked in M-phase by incubation for 18 h in medium containing 20 µg/mL nocodazole. HeLa cells stably expressing CYCA-Luc were blocked in S-phase by incubation in medium containing 5 µg/mL HCPT for 48 h. Cells were trypsinized, washed twice with PBS, and fixed in 70% ethanol at 4°C for at least 1 h. Fixed cells were centrifuged and resuspended in 50 µg/mL RNase and 10 µg/mL propidium iodide for DNA staining at 37°C for 30 min. DNA content was measured by flow cytometry (BD FACS Calibur). DNA histograms were obtained using ModFit LT 3.1 (Verity Software House).

### Cell Extract Luciferase Assay

Luciferase activity in cell extracts was assessed using the Promega Luciferase Assay System according to the manufacturer instructions (Promega). Briefly, cells cultivated and treated with appropriate reagents were washed with PBS and lysed with passive lysis buffer at room temperature. For each assay, 10 µL of whole cell lysate was used for luciferase measure after addition of 100 µL substrate with Lumat LB9507 luminometer. Photon emission was measured for a period of 10 seconds with a delay time of 2 seconds. Luciferase values for stable cell lines were normalized to total protein concentration.

### Western Blot Analysis

Proteins from cultured cells were extracted by lysis in RLB buffer (Promega) and quantitated by BCA protein assay (Pierce Biotechnology). Equal amounts of protein per lane were subjected to SDS-PAGE, transferred to nitrocellulose membranes, and immunoblotted with antibodies against cyclinA2, β-Actin, luciferase, and Cdc20. Protein bands were developed using horseradish peroxidase-conjugated secondary antibodies (Santa Cruz Biotechnology) and developed using ECL detection reagents (Pierce Biotechnology) according to the manufacturer’s instructions.

### Tumor Xenografts

Approximately 1×10^7^ polyclonal HeLa cells producing Luc or monoclonal HeLa cells producing CYCA-Luc in 0.2 mL PBS were injected subcutaneously into sites on each flank of BALB/C nude mice under anesthesia (isoflurane, Abbott Laboratories).

### *In vitro and in vivo* Bioluminescent Imaging

For *in vitro* bioluminescence imaging studies, D-Luciferin was added to tissue culture medium to a final concentration of 150 µg/mL. Five minutes later, photons were counted using the Xenogen IVIS Lumina imaging system (Caliper). Data were analyzed using Living Image software (version 2.6). For *in vivo* studies, at 48 h after intraperitoneal administration of HCPT (0, 1, 5, and 30 mg/kg), mice were administered D-Luciferin (150 mg/kg), by intraperitoneal injection. The anesthetized mice were placed onto warmed stage inside the light-tight IVIS box. In this study, mice were imaged ten minutes after D-Luciferin injection to ensure consistent photon flux emitted during the oxidation of the substrate. The IVIS camera system was used to visualize tumors, and photon measurement was defined around the tumor area and quantified using Living Image software (version 2.6).

### Immunohistochemical Analyses

Mice were euthanized and tumor tissues collected 48 h after intraperitoneal administration of 30 mg/kg HCPT. Tumor tissues were fixed overnight in freshly prepared 4% paraformaldehyde in PBS. Details of the immunohistochemical staining procedures using the SABC method were as previously reported [16]. The expression of cyclin A2 and p27 in tumors was detected using goat polyclonal antibody for cyclin A2 (R & D, 1∶200 dilution) and goat monoclonal antibody for p27 (R & D, 1∶200 dilution). The negative control was normal rabbit IgG-B (Cell Signaling Technology), and positive control slides were included with each assay.

### Statistical Analysis

For imaging data analysis, we calculated the ratio of the intensity of CYCA-Luc luminescence after treatment compared with that of CYCA-Luc before treatment. To control for mouse-to-mouse variability, the CYCA-Luc ratio for each mouse was normalized by dividing by the before/after treatment ratio of luciferase intensity for that mouse. Statistical significance was assessed using Student’s t-test, under the assumption of a normal distribution of the normalized ratios with an estimate of variance. Statistical significance was defined at the standard 5% level.

## Results

### Expression of The CYCA-Luc Fusion Protein in Mammalian Cells

**Figure 1:**
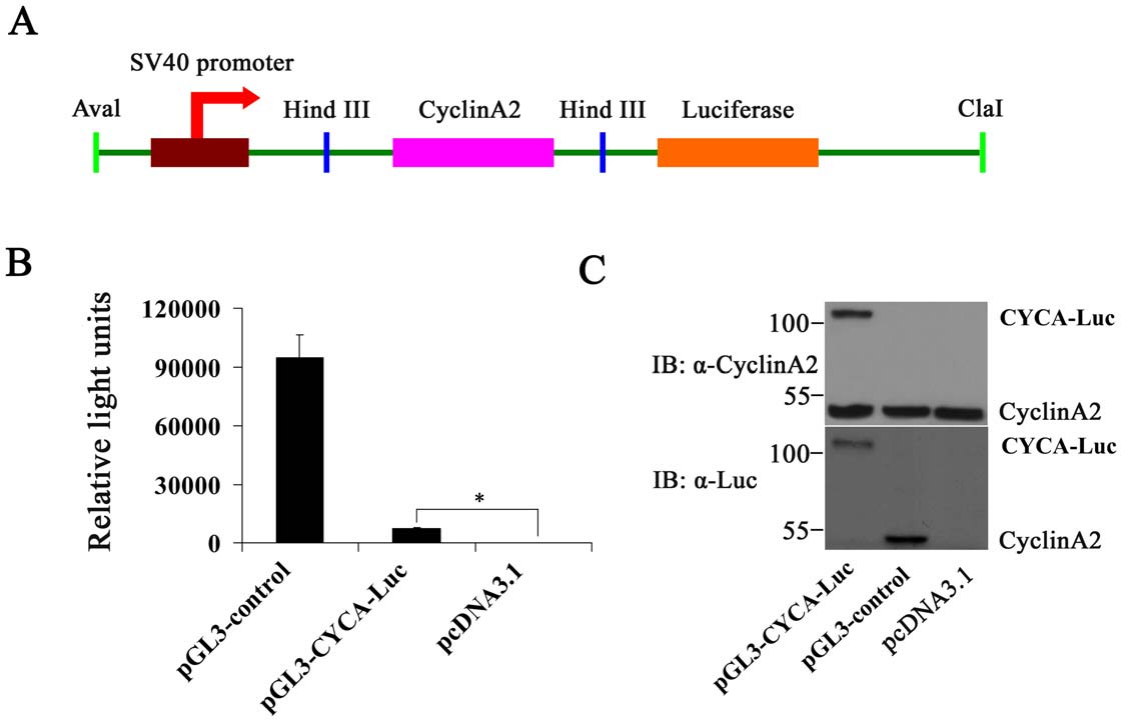
Luciferase activity or immunoblot analysis in U2OS cells following short-term transfection of the wild-type luciferase (Luc) and CYCA-Luc. (**A**) Schematic diagrams of cyclinA2-luciferase constructs. The recombinant plasmid pGL3-CYCA-Luc encoding CYCA-Luc fusion protein contained the cyclin A2 gene fused in-frame at the N termini of the luciferase gene. (**B, C**) U2OS cells were placed into wells of a 6 well plate and transfected using the Lipofectamine 2000 with equal concentrations of plasmid DNA encoding either wild-type luc or CYCA-Luc chimera; pcDNA3.1 plasmid was used as a negative control. Transfected cells were cultured for 48 h, lysed, and cell extracts were assayed for luciferase activity (**B**), or were immunoblotted with the indicated antibodies (**C**). For normalization of CYCA-Luc activity or Luc, the signal (1 µg protein) for control was set to 1. This experiment was repeated three times (n = 3); error bars indicate standard error; *, p<0.05 compared with control.

An expression plasmid (pGL3-CYCA-Luc) encoding full-length cyclin A2 fused in frame to the N-terminus of firefly luciferase under control of the SV40 promoter was constructed (Figure 1A). In order to determine if the fusion gene was effectively translated into a protein, it was expressed in U2OS cells by transient transfection. As a control, the wild-type luciferase gene was transfected into U2OS cells using the same promoter and the same vector (pGL3-control). Substantial luciferase activity was detected in cells transfected either with pGL3-CYCA-Luc or with the wild-type control in protein extracts of transfected cells using similar amounts of plasmids and similar total protein concentrations (Figure 1B). Simultaneously, the CYCA-Luc fusion protein and luciferase (Luc) protein were detected by immunoblot analysis (Figure 1C). These results demonstrated that the CYCA-Luc fusion protein was expressed correctly in mammalian cells.

### CYCA-Luc Fusion Protein is Regulated in a Cell Cycle Dependent Manner

**Figure 2:**
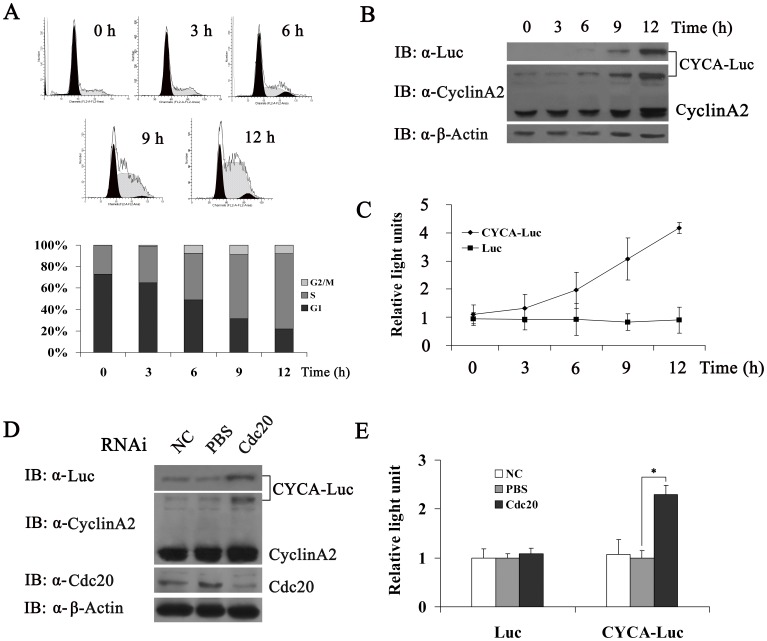
CYCA-Luc mimics cyclin A2 with respect to regulation by the cell cycle. (**A**) U2OS-CYCA-Luc cells were arrested in S-phase by growth in 0.2 mM mimosine. At 0, 3, 6, 9, and 12 h after removal of mimosine, cells were analyzed for DNA content by FACS after propidium iodide staining, or were lysed. (**B, C**) Cell extracts were analyzed by immunoblotting (**B**) or assayed for luciferase activity (**C**). (**D, E**) Immunoblot analysis of U2OS-CYCA-Luc cells (**D**), and luciferase activity in U2OS-CYCA-Luc cells or U2OS-Luc cells (**E**) transfected with Cdc20 or scrambled (negative control) siRNA. For normalization of luciferase or CYCA-Luc activity, the signal for untreated cells was set to 1. This experiment was repeated three times (n = 3). Error bars indicate standard error; *, p<0.05 compared with PBS.

**Figure 3:**
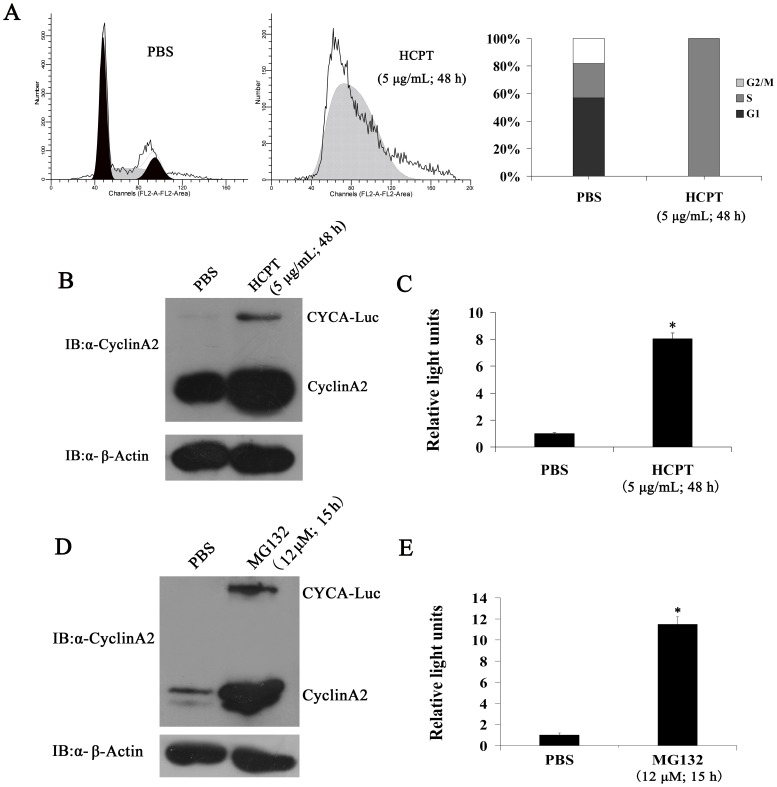
CYCA-Luc accumulates in response to S-phase blockage, and is degraded via the ubiquitin-proteasome pathway in HeLa-CYCA-Luc cells. (**A**) After treatment with 5 µg/mL HCPT for 48 h, HeLa-CYCA-Luc cells were analyzed for DNA content by FACS after propidium iodide staining or were lysed. (**B, C**) Cell extracts were analyzed by immunoblotting (**B**) or assayed for luciferase activity (**C**). After treatment with 12 µmol MG132 for 15 h, HeLa-CYCA-Luc cells were lysed, and cell lysates were analyzed by immunoblotting (**D**) or assayed for luciferase activity (**E**). For normalization of luciferase or CYCA-Luc activity, the signal for untreated cells was set to 1. This experiment was repeated three times (n = 3). Error bars indicate standard error; *, p<0.05 compared with PBS.

We next tested whether CYCA-Luc fusion protein levels might be regulated by the cell cycle in polyclonal U2OS cells producing CYCA-Luc (designated U2OS-CYCA-Luc cells). Cells were synchronized at the G1-S boundary using a mimosine block. Cells were collected for analysis of relative luciferase activity and immunoblot at various time points post-release. As expected, exogenous CYCA-Luc and endogenous cyclin A2 were low in cells arrested at the G_1_-S boundary, and increased steadily as cells entered S-phase after mimosine release, whereas luciferase activity did not change significantly in polyclonal U2OS cells producing Luc (designated U2OS-Luc cells) (Figure 2A–C). Conversely, CYCA-Luc and cyclin A2 were decreased in cells arrested at M-phase by nocodazole (Figure S1). Previous research showed that APC/C^Cdc20^ mediates degradation of cyclinA2 [15]. To confirm that APC/C^Cdc20^ was also responsible for CYCA-Luc degradation, Cdc20 was depleted from U2OS-CYCA-Luc cells using siRNA. Forty-eight hours after siRNA transfection, CYCA-Luc expression significantly increased as compared with mock transfected cells. Induction of CYCA-Luc was demonstrable in immunoblot assays using antibody to luciferase or cyclin A2, as well as in luciferase activity assays. Immunoblotting demonstrated that Cdc20 was effectively depleted by siRNA (Figure 2D, E). These results also showed that treatment with MG132, which is a known proteasome inhibitor, significantly increased CYCA-Luc levels and luciferase activity (Figure S2). These results demonstrated that destruction of CYCA-Luc was mediated by the proteasome and was dependent on APC/C^Cdc20^ ubiquitin ligase. Furthermore, our results also indicated that CYCA-Luc fusion protein levels were regulated by the cell cycle in monoclonal HeLa cells producing CYCA-Luc (designated HeLa -CYCA-Luc cells) (Figure 3).

### CYCA-Luc Accumulates in Response to S-phase Blockage

**Figure 4:**
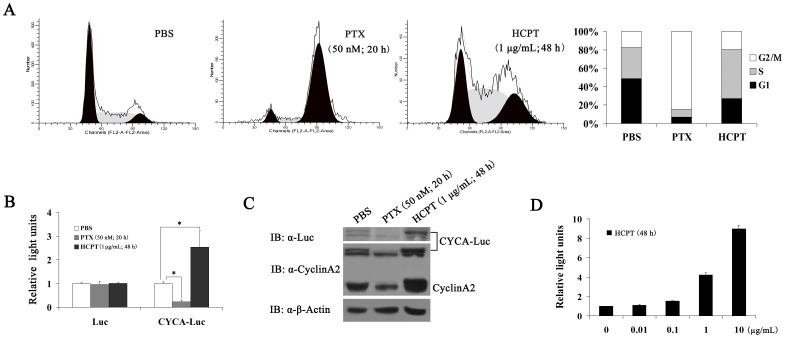
CYCA-Luc accumulates in response to S-phase-specific drug. (**A**) Proportion of cells accumulated in S-phase following S-phase-specific drug (HCPT) treatment or M-phase-specific drug (PTX) treatment. U2OS-CYCA-Luc cells were analyzed for DNA content by FACS after propidium iodide staining, or were lysed, after treatment with PBS (20 h), HCPT (1 µg/mL; 48 h), or PTX (50 nM; 20 h). (**B, C**) Cell extracts were analyzed by immunoblotting (**B**) or assayed for luciferase activity (**C**). (**D**) After treatment with HCPT (0, 0.01, 0.1, 1 and 10 µg/mL) for 48 h, HeLa-CYCA-Luc cells were lysed, and cell lysates were assayed for luciferase activity. For normalization of CYCA-Luc activity or Luc, the signal (1 µg protein) for untreated cells was set to 1. This experiment was repeated three times (n = 3); error bars indicate standard error; *, p<0.05 compared with PBS.

As discussed earlier, HCPT is a natural derivative of camptothecin (CPT), and it is a typical S-phase-specific drug; it can arrest cell cycle in S-phase by selectively inhibiting topoisomerase I (Top I) [17]. In this assay, therefore, we examined whether CYCA-Luc activity could be used to monitor the activity of an S-phase-specific drug (HCPT) *in vitro*. First, we examined the effect of HCPT on the cell cycle of U2OS-CYCA-Luc cells. Flow cytometric analyses with synchronized cells revealed an accumulation of U2OS-CYCA-Luc cells in S-phase after 48 h incubation with HCPT (1 µg/mL). In control analyses, an accumulation of U2OS-CYCA-Luc cells in the M-phase was observed after 20 h incubation with Paclitaxel (*PTX,* 50 nM), an M-phase-specific drug [18] used as a negative control. Compared with the control (without drugs), HCPT treatment resulted in an approximate 2-fold increase in cells in the S-phase; conversely, PTX treatment resulted in an approximate 4 fold decrease in cells in the S-phase (Figure 4A). In the following experiments, we examined the protein expression of exogenous CYCA-Luc or endogenous cyclin A2 and luciferase activity. As expected, treatment with HCPT significantly increased CYCA-Luc levels and luciferase activity, and PTX treatment significantly decreased CYCA-Luc levels and luciferase activity (Figure 4B, C). Furthermore, HCPT also caused a dose-dependent increase in luciferase activity in HeLa-CYCA-Luc cells (Figure 4D). These results demonstrated that HCPT induced S-phase arrest of cell cycle and increased CYCA-Luc expression in cells producing CYCA-Luc under *in vitro* conditions.

### Response of Reporter Cells to Pharmacological Agent

**Figure 5:**
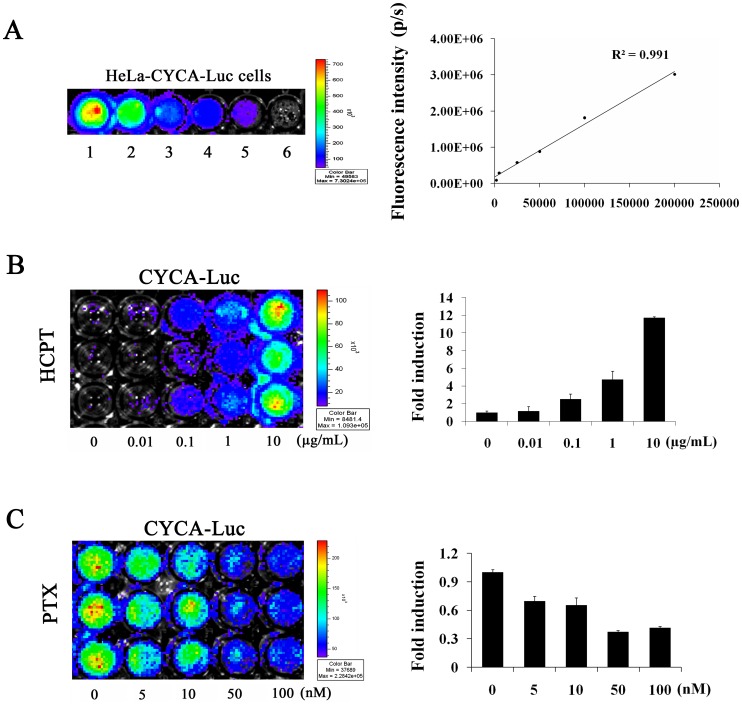
Induction of CYCA-Luc by S-phase-specific drug *in vitro*. (**A**) HeLa-CYCA-Luc cells were serially diluted, placed into wells of a 96 well plate, and immediately imaged using the IVIS Lumina imaging system to obtain FLUX measurements (**left, images**). These data were averaged (n = 3) and used to generate a plot comparing total flux to cell number (**right, graph**). (**B, C**) HeLa-CYCA-Luc cells were placed into wells of a 96 well plate. Images were obtained after 48 h treatment with HCPT (0, 0.01, 0.1, 1, and 10 µg/mL) (**B**), or 20 h treatment with PTX (0, 5, 10, 50 and 100 nM) (**C**). Left, cellular images obtained after treatment with HCPT or PTX. Right, normalized fold induction of CYCA-Luc or Luc treated with the indicated doses of drugs. For normalization of luciferase or CYCA-Luc activity, the signal for untreated cells was set to 1. Quantitative data represent the mean ± standard error (n = 3 per group).

Bioluminescence imaging (BLI) uses light measurements (FLUX) to monitor tumor progression and drug responses. FLUX data represents the number of luciferase positive cells in a given region of interest [19]. In order to correlate FLUX values determined *in vivo* to the estimated number of viable cells, dilutions of HeLa-CYCA-Luc cells derived from culture were prepared (0 to 2.0×10^5^ cells) and placed in wells of a 96-well plate. D-Luciferin was added directly to the cell culture medium and the plate was then imaged using the Xenogen IVIS Lumina imaging system. FLUX measurements were determined and plotted against the number of cells plated per well to generate a curve relating FLUX to cell number (Figure 5A). After performing this experiment, we examined the effect of HCPT on CYCA-Luc activity by imaging *in cellulo*. As expected, HCPT caused a dose-dependent increase in luciferase signals (Figure 5B), whereas PTX caused a dose-dependent decrease in luciferase signals in HeLa-CYCA-Luc cells (Figure 5C). However, treatment with HCPT had no significant effect on wild-type luciferase signals in polyclonal HeLa cells producing Luc (HeLa-Luc cells) (Figure S3). Furthermore, HCPT also caused a dose-dependent increase in luciferase signals in U2OS-CYCA-Luc cells (Figure S4). These results demonstrated that the CYCLA-Luc fusion protein was suitable for monitoring cancer cells in response to S-phase-specific drug, HCPT treatment under *in vitro* conditions.

### Imaging S-phase-specific Drug Pharmacodynamics *in vivo*

**Figure 6:**
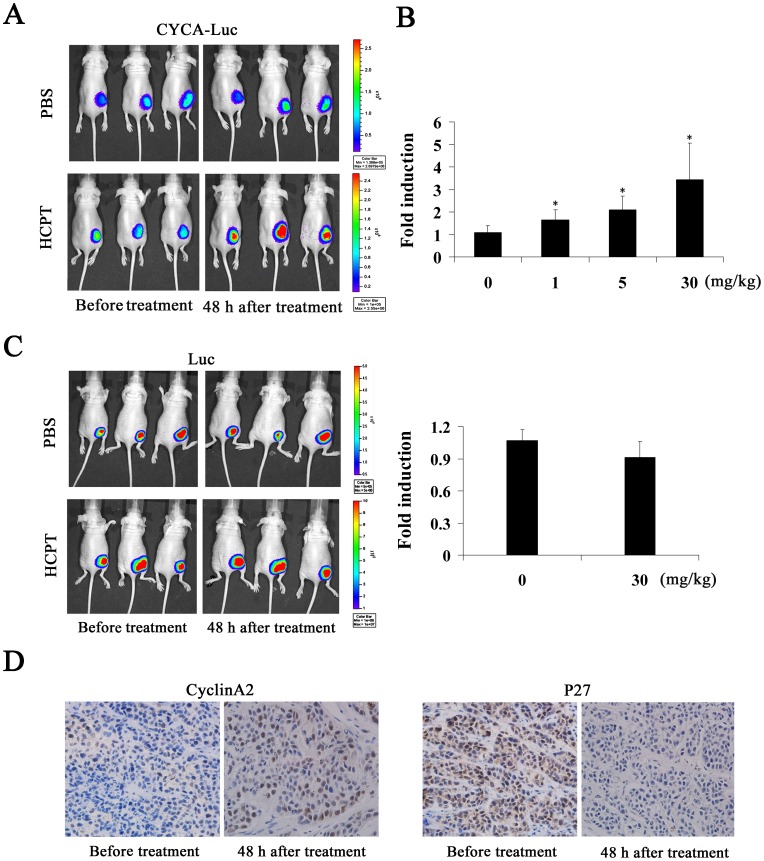
Monitoring S-phase-specific drug *in vivo* using bioluminescent imaging. (**A, C**) 1×10^7^ HeLa-CYCA-Luc cells or HeLa-Luc cells in 0.2 mL PBS were injected subcutaneously into each flank of BALB/C nude mice under anesthesia (isoflurane). (**A**) Left, Bioluminescent images were obtained 3 weeks later, when tumors of comparable size (∼5 mm) had formed bilaterally. Right, repeat images were obtained 48 h after intraperitoneal injection of HCPT (30 mg/kg). (**B**) Normalized fold induction of CYCA-Luc in mice treated with the indicated doses of HCPT (four mice per treatment group). (**C**) Normalized fold induction of Luc in mice treated with the indicated doses of HCPT (four mice per treatment group) and imaged as in **A**. (**B, C**) Fold induction was calculated as CYCA-Luc/Luc_post-treatment_ ÷ CYCA-Luc/Luc_pretreatment_. Error bars indicate standard error; *, p<0.05 compared with PBS. (**D**) Representative immunohistochemical images of cyclin A2 and p27. Tumor sections were obtained 48 h after initial injection of 30 mg/kg HCPT (n = 4 per group). The representative images were taken at an original magnification of 200×.

To validate these findings in an *in vivo* model, we next evaluated the effect of HCPT on bioluminescence activity in a subcutaneous mouse tumor model. First, HeLa-CYCA-Luc cells or HeLa-Luc cells were implanted subcutaneously in nude mice. Three weeks after implant, the mice were imaged using the Xenogen IVIS Lumina imaging system. D-Luciferin (150 mg/kg) was then injected into the intraperitoneal cavity of each mouse. The bioluminescence signal reached plateau phase at the 10 min time point (Figure S5); therefore, animals were imaged at a standard point (10 min) after D-Luciferin injection. Next, we examined the effect of HCPT on bioluminescence signals from HeLa-CYCA-Luc tumors. As expected, CYCA-Luc activity was significantly induced by HCPT in a dose-dependent manner *in vivo*, but we did not find significant differences in the PBS treatment group (Figure 6A, B). Furthermore, our results also showed that the bioluminescence signals from HeLa-Luc tumors did not change significantly in response to HCPT treatment (Figure 6C). It has been reported that cyclin A2 levels were increased while p27 levels were decreased in progression through G1 to S-phase [20]. In this assay, therefore, the expression of p27 and cyclin A2 was determined by immunohistochemistry. The results showed that HCPT treatment (30 mg/kg) significantly increased cyclin A2 protein levels and significantly decreased p27 protein levels in tumor tissue (Figure 6D). Thus, these results demonstrated that the CYCLA-Luc fusion protein was suitable for monitoring S-phase-specific drug, HCPT treatment in living animals.

## Discussion

Bioluminescence reporter proteins have been widely used in the development of tools for monitoring biological events in living organisms in real time [21]. Many aspects of drug development can be facilitated using bioluminescence reporter proteins as an indicator to discover new targets, identify novel drug candidates, and validate their potency [22], [23]. In the present study, we found that a reporter consisting of cyclin A2 fused to luciferase was responsive to S-phase-specific anti-cancer drug, HCPT in *cellulo* and *in vivo*. Responsiveness to drug activity was validated using the IVIS imaging system. We demonstrated that the CYCA-Luc reporter can provide a pharmacodynamic readout of S-phase-specific anti-cancer drug action, HCPT in animal models.

Due to the rapid progress in the field of cancer chemotherapy and the continued synthesis of agents that arrest the growth of cancer cells through the inhibition of the DNA replication process, there is a compelling need for *in vitro* and *in vivo* models that can be used for rapid and accurate analysis of the molecular actions of these anti-cancer drugs. To date, several techniques have been developed for studying S-phase-specific anti-cancer drug activity. The usefulness of the DNA synthesome as an *in vitro* model system for analyzing the molecular mechanism of S-phase-specific anti-cancer agents has been reported previously [24], [25]. Although these systems proved to be useful in screening S-phase-specific anti-cancer drugs, they have several disadvantages in the context of *in vivo* analysis and sequential monitoring of S-phase-specific drugs activity as compared to the molecular imaging techniques. To circumvent these limitations, in this study, we have developed a reporter (CYCA-Luc) system based on ubiquitin-proteasome dependent degradation, which allows precise monitoring of S-phase in real time *in vivo*. We have tested this reporter for its ability to correctly predict the anticipated effects of S-phase-specific anti-cancer agent, HCPT. Our studies validated the usefulness of the reporter as an *in vivo* model system for analyzing the molecular mechanism of HCPT.

Our experiments *in vitro* showed that the CYCA-Luc fusion protein accumulates during S-phase and is abruptly degraded during G2/M phase similar to endogenous cyclinA2. We showed that APC/C^Cdc20^ mediated degradation of CYCA-Luc. These results suggest that the CYCA-Luc fusion protein is regulated by cell cycle. Moreover, HCPT caused a dose-dependent increase in luciferase signal in various tumor cells (U2OS osteosarcoma, HeLa cervical carcinoma) stably producing CYCA-Luc, but not in cells producing wild-type luciferase. Most importantly, our experiments *in vivo* showed a clear dose-response relationship, with a small response seen with 1 mg/kg treatment and a maximal response observed at 30 mg/kg. The peak luciferase signal was detected at 24 h, and peak levels were detected for up to 48 h. In addition, we also observed that the CYCA-Luc protein did not induce significant cytotoxic effects *in vitro* or *in vivo*. Thus, the development of this reporter opens up the possibility of screening of S-phase-specific anti-cancer drugs, HCPT and its derivatives in cell culture and in live small animal models of cancer.

This bioluminescence reporter is applicable not only to the evaluation of drug efficacy but also to the study of intracellular molecular pathways [26]. For example, in a previous study, a p27-luciferase-expressing tumor cell was used to monitor Cdk2 activity by *in vivo* bioluminescence imaging in hollow fibers [12]. Moreover, a luciferase plasmid driven by an NFκB-responsive element was stably transfected into tumor cells, and the resultant reporter cell line was then evaluated in the hollow fiber model after treatment of the host mice with either LPS or TNF-α [9]. Thus, in addition to pharmacodynamic analysis, this reporter system can be extended to study other physiological processes related to cyclin A2. As previously mentioned, APC/C^Cdc20^ mediates degradation of cyclinA2, and the degradation is spindle-checkpoint independent and thus, it starts as soon as APC/C^Cdc20^ is activated [27]. Therefore, this CYCA-Luc reporter may be used to monitor APC/C^Cdc20^ activity in cultured cells and in living animals. Moreover, the precise timing of cyclin A2 degradation is still unclear; thus, this reporter system provides a useful tool for elucidating the molecular mechanisms of cyclin A2 degradation during mitosis *in vitro* and *in vivo* [28].

In summary, we developed and assessed a fusion protein reporter in cancer cells and living animals. This fusion protein-based reporter has several advantages that make it an ideal *in vivo* model to study S-phase targeted anti-cancer drug action. This fusion reporter system can be used to monitor S-phase of cell cycle, and evaluate pharmacological activity of S-phase targeted anti-cancer drug, HCPT in real time *in vitro* and *in vivo*. However, cyclin A2 is expressed at early S-phase, accumulates throughout S and G2 [29], [30], so, we do not exclude the possibility that this might also be used to reflect G2-phase of cell cycle. It also provides a promising new tool for both *in vitro* high-throughput screening and kinetic analysis and for *in vivo* validation of a relatively large number of drugs in a short period of time. This fusion protein reporter should expedite preclinical drug discovery.

## Supporting Information

Figure S1 CYCA-Luc is decreased in cells arrested at M-phase by nocodazole. (**A**) After treatment with 20 µg/mL nocodazole for 18 h, U2OS-CYCA-Luc cells were analyzed for DNA content by FACS after propidium iodide staining or were lysed. (**B, C**) Cell extracts were analyzed by immunoblotting (**B**) or assayed for luciferase activity (**C**). For normalization of luciferase or CYCA-Luc activity, the signal for untreated cells was set to 1. This experiment was repeated three times (n = 3). Error bars indicate standard error; *, p<0.05 compared with PBS.
(TIF)

Figure S2 Analysis of CYCA-Luc accumulation in cells treated with MG132. After treatment with 1 µmol MG132 for 48 h, U2OS-CYCA-Luc cells were lysed, and cell lysates were analyzed by immunoblotting (**A**) or assayed for luciferase activity (**B**). For normalization of luciferase or CYCA-Luc activity, the signal for untreated cells was set to 1. This experiment was repeated three times (n = 3). Error bars indicate standard error; *, p<0.05 compared with PBS.
(TIF)

Figure S3 Bioluminescent HeLa-Luc cells respond to S-phase-specific drug. HeLa-Luc cells were placed into wells of a 96 well plate. Images were obtained after 48 h treatment with HCPT (0, 0.01, 0.1, 1, and 10 µg/mL). Left, cellular images obtained after treatment with HCPT. Right, normalized fold induction of luciferase signals after treatment with the indicated doses of drugs. Luciferase signal was normalized to a value of 1 for untreated cells. Quantitative data represent the mean ± standard error (n = 3 per group).
(TIF)

Figure S4 Bioluminescent U2OS-CYCA-Luc cells respond to S-phase-specific drug. U2OS-CYCA-Luc cells were placed into wells of a 96 well plate. Images were obtained after 48 h treatment with HCPT (0, 0.001, 0.01, 0.1, and 1 µg/mL). Left, cellular images obtained after treatment with HCPT. Right, normalized fold induction of luciferase signals after treatment with the indicated doses of drugs. Luciferase or CYCA-Luc signal was normalized to a value of 1 for untreated cells. Quantitative data represent the mean ± standard error (n = 3 per group).
(TIF)

Figure 5S Kinetic profile of bioluminescent signal for d-Luciferin administration. Mice were administered D-Luciferin (150 mg/kg) by intraperitoneal injection. Images were obtained at various time points (5, 10, 15, 20, 25, 30, 35, 40, and 45 min) after intraperitoneal injection of D-Luciferin (**left, images**). These data were averaged (n = 3) and used to generate a plot of total flux (**right, graph**).
(TIF)
