# Supplementary figures and images for: Bioluminescence Imaging of DNA Synthetic Phase of Cell Cycle in Living Animals
Source: PLoS One. 2013 Jan 3;8(1):e53291. doi: 10.1371/journal.pone.0053291 (PMC3536746; doi:10.1371/journal.pone.0053291)

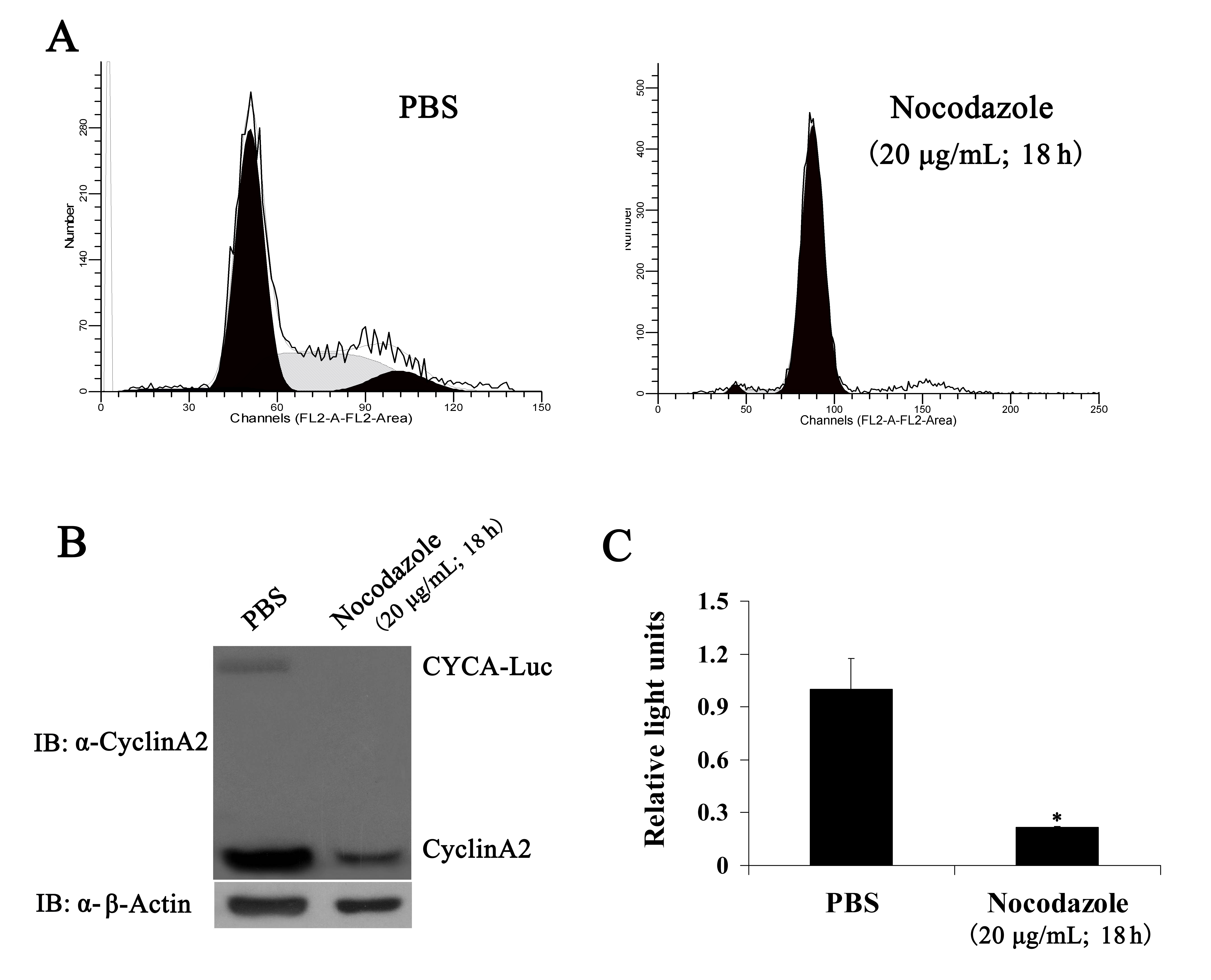

Supplement: Figure S1 — CYCA-Luc is decreased in cells arrested at M-phase by nocodazole. (A) After treatment with 20 µg/mL nocodazole for 18 h, U2OS-CYCA-Luc cells were analyzed for DNA content by FACS after propidium iodide staining or were lysed. (B, C) Cell extracts were analyzed by immunoblotting (B) or assayed for luciferase activity (C). For normalization of luciferase or CYCA-Luc activity, the signal for untreated cells was set to 1. This experiment was repeated three times (n = 3). Error bars indicate standard error; *, p<0.05 compared with PBS. (TIF) [file pone.0053291.s001.tif]

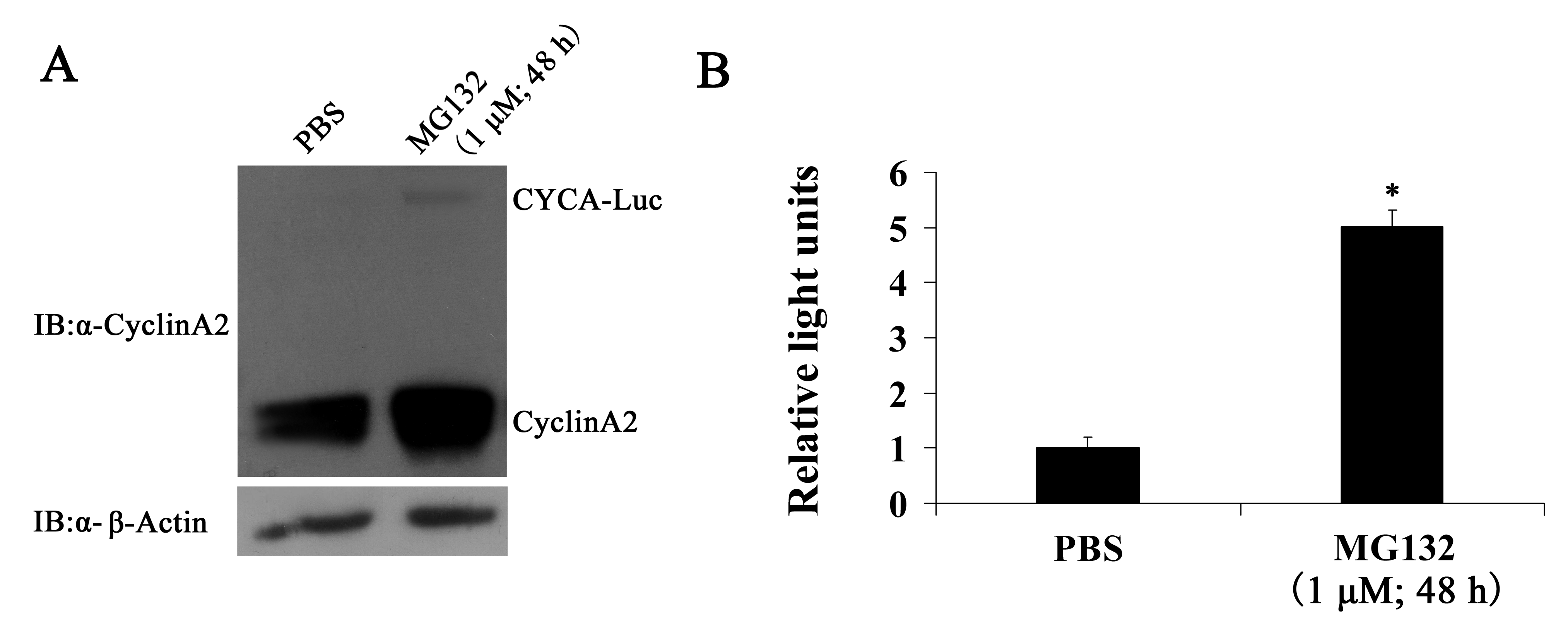

Supplement: Figure S2 — Analysis of CYCA-Luc accumulation in cells treated with MG132. After treatment with 1 µmol MG132 for 48 h, U2OS-CYCA-Luc cells were lysed, and cell lysates were analyzed by immunoblotting (A) or assayed for luciferase activity (B). For normalization of luciferase or CYCA-Luc activity, the signal for untreated cells was set to 1. This experiment was repeated three times (n = 3). Error bars indicate standard error; *, p<0.05 compared with PBS. (TIF) [file pone.0053291.s002.tif]

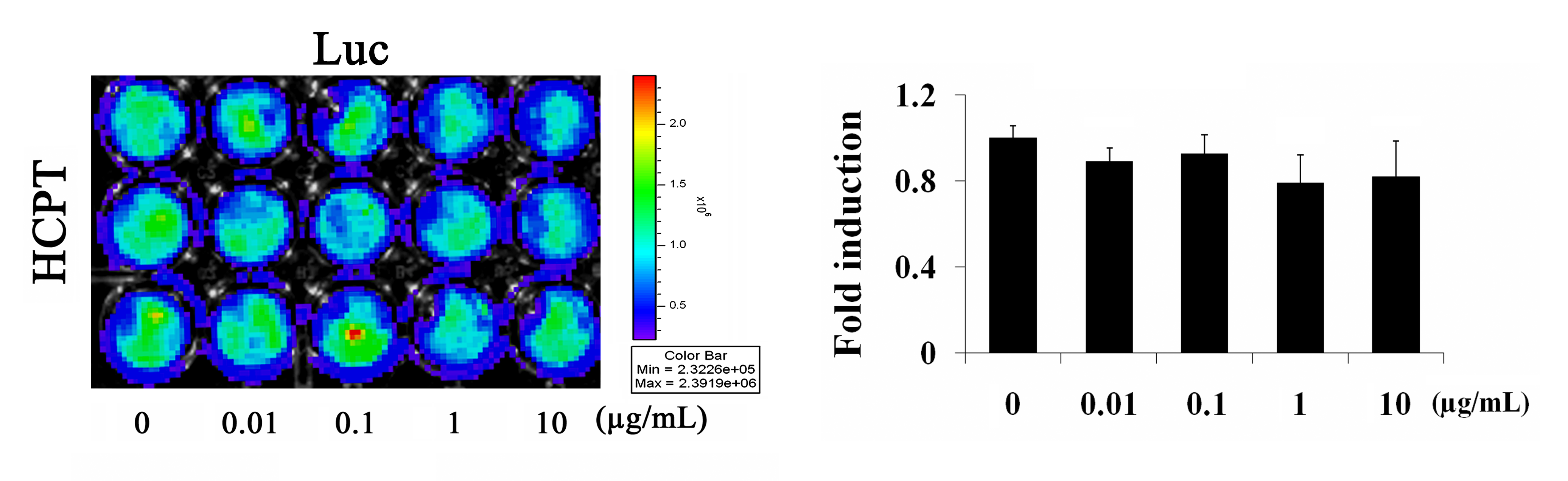

Supplement: Figure S3 — Bioluminescent HeLa-Luc cells respond to S-phase-specific drug. HeLa-Luc cells were placed into wells of a 96 well plate. Images were obtained after 48 h treatment with HCPT (0, 0.01, 0.1, 1, and 10 µg/mL). Left, cellular images obtained after treatment with HCPT. Right, normalized fold induction of luciferase signals after treatment with the indicated doses of drugs. Luciferase signal was normalized to a value of 1 for untreated cells. Quantitative data represent the mean ± standard error (n = 3 per group). (TIF) [file pone.0053291.s003.tif]

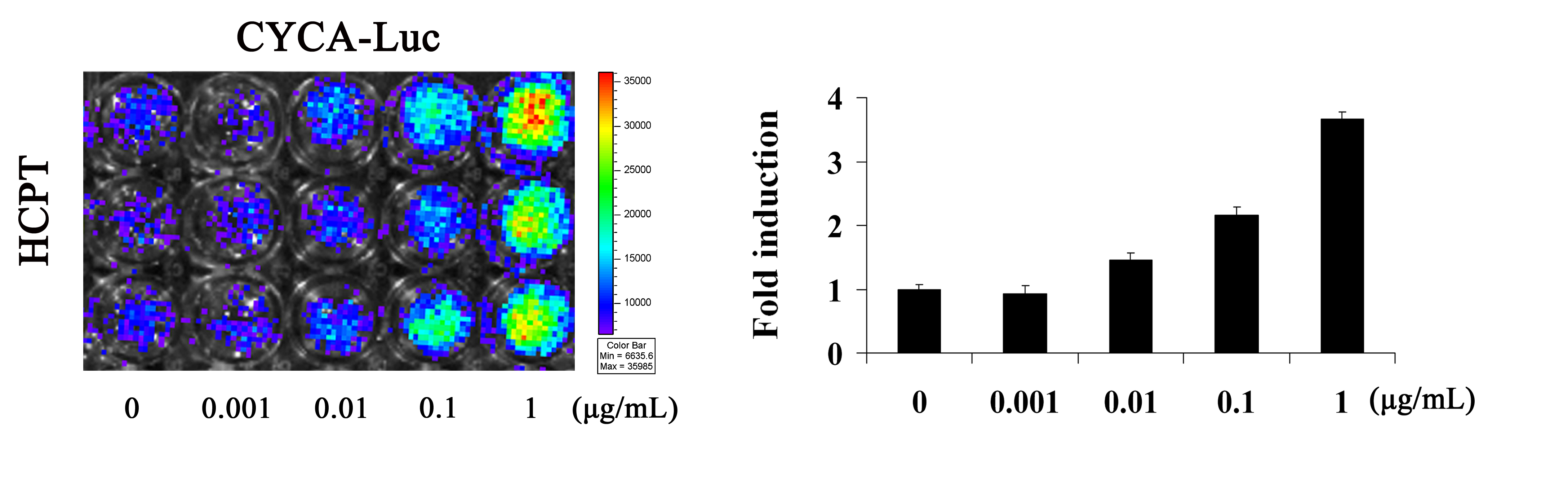

Supplement: Figure S4 — Bioluminescent U2OS-CYCA-Luc cells respond to S-phase-specific drug. U2OS-CYCA-Luc cells were placed into wells of a 96 well plate. Images were obtained after 48 h treatment with HCPT (0, 0.001, 0.01, 0.1, and 1 µg/mL). Left, cellular images obtained after treatment with HCPT. Right, normalized fold induction of luciferase signals after treatment with the indicated doses of drugs. Luciferase or CYCA-Luc signal was normalized to a value of 1 for untreated cells. Quantitative data represent the mean ± standard error (n = 3 per group). (TIF) [file pone.0053291.s004.tif]

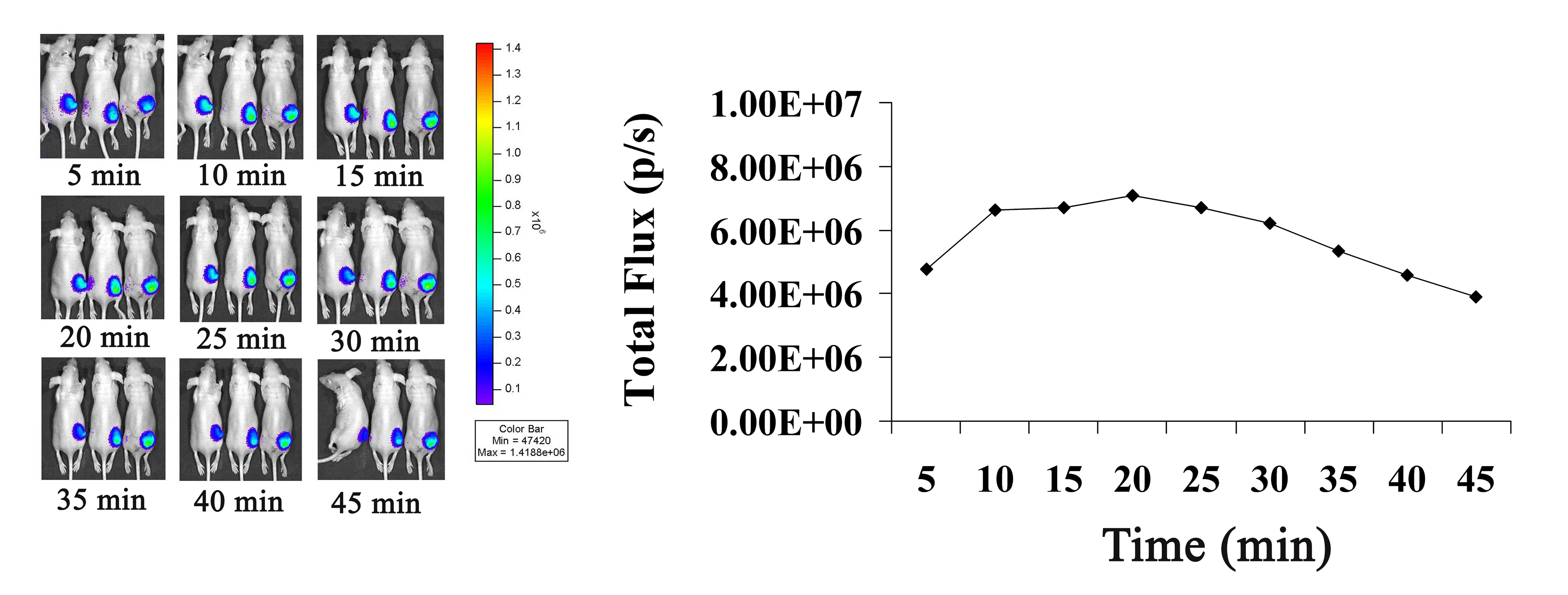

Supplement: Figure 5S — Kinetic profile of bioluminescent signal for d-Luciferin administration. Mice were administered D-Luciferin (150 mg/kg) by intraperitoneal injection. Images were obtained at various time points (5, 10, 15, 20, 25, 30, 35, 40, and 45 min) after intraperitoneal injection of D-Luciferin (left, images). These data were averaged (n = 3) and used to generate a plot of total flux (right, graph). (TIF) [file pone.0053291.s005.tif]
